# Knowledge and Attitudes about Antibiotics and Antibiotic Resistance of 2404 UK Healthcare Workers

**DOI:** 10.3390/antibiotics11081133

**Published:** 2022-08-21

**Authors:** Diane Ashiru-Oredope, Ella Casale, Eleanor Harvey, Eno Umoh, Sagar Vasandani, Jacqui Reilly, Susan Hopkins

**Affiliations:** 1 HCAI, Fungal, AMR, AMU & Sepsis Division, United Kingdom Health Security Agency, London SW1P 3JR, UK; 2 NHS National Services Scotland, Edinburgh EH12 9EB, UK; 3 Department of Nursing and Community Health, Glasgow Caledonian University, Glasgow G4 0BA, UK

**Keywords:** anti-infective, antimicrobial, antimicrobial resistance, behaviour change, healthcare workers, antimicrobial stewardship

## Abstract

Background: Using the COM-B model as a framework, an EU-wide survey aimed to ascertain multidisciplinary healthcare workers’ (HCWs’) knowledge, attitudes and behaviours towards antibiotics, antibiotic use and antibiotic resistance. The UK findings are presented here. Methods: A 43-item questionnaire was developed through a two-round modified Delphi consensus process. The UK target quota was 1315 respondents. Results: In total, 2404 participants responded. The highest proportion were nursing and midwifery professionals (42%), pharmacists (23%) and medical doctors (18%). HCWs correctly answered that antibiotics are not effective against viruses (97%), they have associated side effects (97%), unnecessary use makes antibiotics ineffective (97%) and healthy people can carry antibiotic-resistant bacteria (90%). However, fewer than 80% correctly answered that using antibiotics increases a patient’s risk of antimicrobial resistant infection or that resistant bacteria can spread from person to person. Whilst the majority of HCWs (81%) agreed there is a connection between their antibiotic prescribing behaviour and the spread of antibiotic-resistant bacteria, only 64% felt that they have a key role in controlling antibiotic resistance. The top three barriers to providing advice or resources were lack of resources (19%), insufficient time (11%) and the patient being uninterested in the information (7%). Approximately 35% of UK respondents who were prescribers prescribed an antibiotic at least once in the previous week to responding to the survey due to a fear of patient deterioration or complications. Conclusion: These findings highlight that a multifaceted approach to tackling the barriers to prudent antibiotic use in the UK is required and provides evidence for guiding targeted policy, intervention development and future research. Education and training should focus on patient communication, information on spreading resistant bacteria and increased risk for individuals.

## 1. Introduction

In England, antimicrobial resistant infections increased between 2016 and 2019, peaking at an estimated 65,192 infections in 2019 [1]. Nationally and internationally, several policy papers have emphasised the need to address this globally recognised, complex threat to human health [2,3,4,5]. The UK’s five-year National Action Plan (NAP) to tackle antimicrobial resistance (AMR) emphasises the importance of reducing our need for, and exposure to, antimicrobials, and optimising the use of these life-saving drugs in humans and animals. Healthcare workers’ (HCWs’) practices impact infections and antimicrobial use; therefore, they are vital in our efforts to tackle AMR. Moreover, HCWs play an instrumental role in implementing antimicrobial stewardship interventions, highlighting the necessity of understanding their knowledge, attitudes and behaviour towards antibiotics, antibiotic use and AMR.

To support HCWs in tackling AMR, Public Health England (PHE) published a resources toolkit aimed at healthcare workers in 2014, which outlines key actions HCWs can take to help combat AMR [6]. Studies and public health campaigns that investigate and target the general public’s knowledge and understanding of antibiotics and antibiotic resistance have been conducted [7,8]. The literature reports good basic knowledge with areas for improvement, such as targeting the misunderstanding that these drugs can be used to treat viral infections [9,10]. Evidence also exists for a subset of medical prescribers and nurses. Earlier studies have reported good knowledge of antibiotics among nurse practitioners and their intention to prescribe antibiotics appropriately [11,12]. Factors impacting nurse and physician prescribing have included patient pressure, fear, complacency and time [12,13]. Although there is some evidence for medical prescribers and nurses, a gap remains in the literature regarding multidisciplinary HCWs’ knowledge, attitudes and behaviours towards this topic [11,12,13,14,15].

The European Centre for Disease Prevention and Control (ECDC) funded the first multiprofessional study across 30 European Union (EU) and European Economic Area (EEA) countries to assess HCWs’ baseline knowledge, attitudes and behaviours surrounding antibiotics in 2019. This paper presents the results for the United Kingdom and aims to:

- Assess the knowledge, attitudes and behaviour of UK HCWs (including prescribers) in regard to antibiotics and antibiotic use and resistance;
- Determine baseline data from a pre-COVID-19 pandemic landscape to allow for the future comparison of the impact of the pandemic on antimicrobial awareness and response and inform policy and interventions;
- Support the evaluation of UK communication campaigns (UK National Action Plan (NAP), Antibiotic Guardian and the Keep Antibiotics Working (KAW) campaign) and international awareness activities (World Antimicrobial Awareness Week (WAAW) and European Antibiotic Awareness Day (EAAD)) which have been targeting HCWs for over a decade.

## 2. Materials and Methods

This study was part of a wider 2019 ECDC-funded survey across 30 EU/EEA countries. The full methodology is described elsewhere [16,17]. Using the COM-B model of behaviour change as a framework, this study sought to understand the capabilities (C), opportunities (O) and motivations (M) which enable prudent behaviour (B) about antibiotic use amongst healthcare workers in the UK [18].

The COM-B model was selected to underpin the questionnaire and analysis because it is a comprehensive synthesis and integration of at least 19 models of behaviour change [19]. The COM-B model proposes that behaviour is an interaction involving three essential components: the ‘capability’ (C) to perform the behaviour and the ‘opportunity’ (O) and ‘motivation’ (M) to carry out the behaviour. Studies of the model have shown that to affect behaviour change, any introduced interventions must influence as least one of these three components, so as to reshape behaviour and minimise setbacks [18].

The final questionnaire is available in the Supplementary Materials. Perceived and actual capability were measured to assess HCWs’ knowledge. Perceived capability was assessed via self-reported understanding of antibiotic use and antibiotic resistance; participants were asked 3 questions about their own understanding. Actual capability was assessed via a 7-question knowledge test, with additional questions to assess one health and hand hygiene topics. Participants were asked 3 opportunity statements to assess whether they have the opportunity to contribute to tackling AMR; these focused on access to guidelines and materials and whether they were able to provide advice to patients. Motivation statements were utilised to assess attitudes towards antibiotics and their use. Behaviour investigated included the frequency of prescribing and whether HCWs gave out resources and advice.

A Project Advisory Group (PAG) consisting of 87 nominated individuals across 51 organisations and countries within the EU and EEA was established in 2018, of these 2 were from the UK. Following a systematic review of the literature, the PAG participated in a two-round Delphi consensus process to develop the questionnaire tool. The final 43-item questionnaire was developed following a pilot of the survey across the EU/EEA, translation into 25 European languages and validation.

PAG members disseminated the validated online survey to HCWs in each of the 30 included countries. Multiple dissemination routes were used in the UK including email cascade via national AMR groups with representation from multidisciplinary professional groups asking them to cascade wider and social media, using the hashtag #ECDCAntibioticSurvey on Twitter. The information provided to survey participants is available in the Supplementary Materials. The questionnaire was live for 6 weeks, from 28 January to 4 March 2019. Participation was voluntary. Data were collected anonymously, with the option to submit contact details. Public Health England was responsible for housing all data securely and as per the General Data Protection Regulation 2016/679.

The statements used a five-point Likert score; for the purposes of this report, agreed and strongly agreed were merged in the text and reported as agreed.

The minimum survey sample size was obtained via quota sampling with the aim of generating a representative sample from different healthcare worker groups and countries and capturing different demographics, specialties, levels of experience and sectors of healthcare [16]. The sample size for the UK was calculated by determining 0.2% of practicing physicians (365/182,534), dentists (70/34,867) and pharmacists (113/56,542) and 0.1% of nursing professionals (548/548,291) available through EU/EEA healthcare personnel statistics of healthcare workers [17,20].

Data were analysed using Microsoft^®^ Excel (2010) and STATA release 15. Descriptive statistical analysis was undertaken to analyse HCW capability, opportunity, motivation and behaviour.

The results of the UK study were shared with thirty-four attendees across the UK devolved administrations in June 2020 to inform recommendations.

Ethical statement: The data for this study were extracted from and comply with the ethical statement for the research undertaken by Ashiru-Oredope et al. [16]. Informed consent was provided prior to participation. The original study was part of an evaluation of the EU EAAD communications campaign which commenced in 2008 and included significant input in development and distribution from the PAG. The PAG officially represented all participating countries and European professional groups and organisations.

## 3. Results

### 3.1. Demographics

In the EU/EEA multicountry study, 18,365 HCWs responded to the online survey. Of these, 2404 participants were from the UK, equating to 13% of the EU/EEA total and the largest proportion among all 30 countries involved. The highest number of responses was collected from England (51%), followed by Scotland (40%), Wales (7%) and Northern Ireland (2%) (Supplementary Table S1). The highest number of participants stratified by English region were from the North West (23%), London (15%) and the South West (12%) (Supplementary Figure S1).

The UK exceeded its quota sample size by 979 responses, achieving 174% of the desired target (Supplementary Table S2). In England, Scotland and Wales, nursing professionals (nurses, nursing assistants and midwives) had the highest response rate (40%), followed by pharmacists (22%) and medical doctors (18%) (Supplementary Table S3). In Northern Ireland, pharmacists constituted the largest respondent group, followed by medical professionals and scientists. Healthcare students were not included in this sample.

Similarly to the EU/EEA survey results, most UK participants were female (77%), 21% were male and 1% preferred not to state their sex (Supplementary Table S4). UK respondents were mostly aged between 36 and 55 years (59%), 23% were under 36 years and 17% over 55 years. Respondents predominantly practised in hospitals (58%) or the community (31%).

In total, 24% of UK respondents used Facebook and 23% used Twitter for professional purposes. (Supplementary Table S5). Almost half of UK respondents (44%) did not use any social media for professional purposes.

### 3.2. Perceived Capability

**Figure 1:**
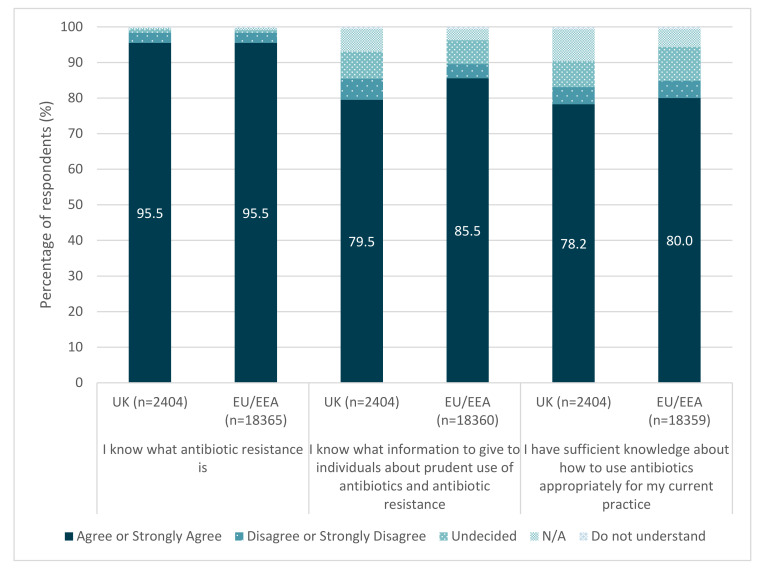
UK respondents’ perceived capability.

The majority (96%) of UK respondents agreed or strongly agreed that they ‘know what antibiotic resistance is’ (Figure 1). Over three-quarters (78%) agreed or strongly agreed that they had sufficient knowledge on using antibiotics in their practice, and 80% agreed or strongly agreed that they know what information to provide to their patients on the prudent use of antibiotics and antibiotic resistance.

### 3.3. Actual Capability Assessed by Seven Knowledge Test Questions

**Figure 2:**
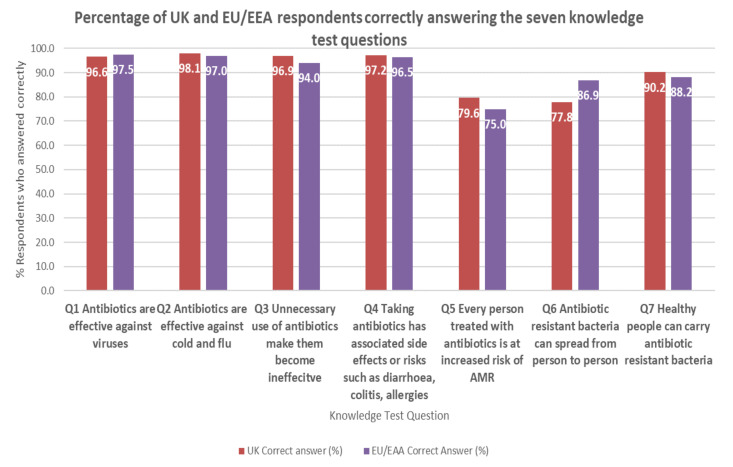
Percentage of UK (*n* = 2403) and EU/EEA respondents (*n* = 18,348) correctly answering the seven knowledge test questions.

The highest proportion of correct answers in the UK were given to the true or false questions: ‘Antibiotics are effective against cold and flu’ (answer = false) (98% false and 2% true); ‘Taking antibiotics has associated side effects or risks such as diarrhoea, colitis, allergies’ (answer = true) (97% true and 3% false); and ‘Unnecessary use of antibiotics make them become ineffective’ (answer = true) (97% true and 3% false) (Figure 2). Over 90% of HCWs knew that healthy people can carry antibiotic-resistant bacteria.

The question that was answered incorrectly by the most respondents was ‘Antibiotic resistant bacteria can spread from person to person’ (answer = true) (22% of respondents incorrectly answered false), which was higher than the percentage of incorrect answers from the EU/EEA respondents (13%) for that same question. The second statement with the most incorrect answers in the UK was ‘Every person treated with antibiotics is at an increased risk of antibiotic resistant infection’ (answer = true) (20% incorrectly answered false); this question had a higher rate of incorrect answers from the EU/EEA respondents (25%).

In total, 59.4% of UK participants were able to answer all seven knowledge questions correctly. The EU/EEA average was 58%.

The answers to the knowledge test questions varied across professional groups. Medical doctors demonstrated the best knowledge of AMR (80% answered all questions correctly), followed by pharmacists (74%), dentists (68%) and scientists (62%) (Supplementary Figure S2). Only medical doctors had all the representatives scoring a minimum of five out of seven correct answers. Questions 5 and 6 were answered most correctly by medical doctors (86% and 96%, respectively (Supplementary Figure S3).

The percentage of respondents answering all seven questions correctly varied across the devolved administrations (54–80%). Northern Ireland had the highest proportion of respondents answering all seven questions correctly (80%, *n* = 41) (Supplementary Table S6). Across the EU/EEA, no country had 100% of respondents achieve 7/7 in the knowledge score.

Less than a quarter (23%) of UK participants correctly answered that the use of antibiotics to stimulate growth in farm animals is illegal in the EU (Supplementary Table S7). Almost half of respondents said they could list the WHO’s five moments of hand hygiene (Supplementary Table S8). 

### 3.4. Opportunities

Four out of five (80%) UK respondents stated having easy access to guidelines, compared to 75% of EU/EEA participants (Table 1). Only 62% of practitioners in the UK felt they have good opportunities to provide advice on prudent antibiotic use to individuals, compared to 72% of EU/EEA participants.

### 3.5. Motivation/Attitude towards Antibiotic Resistance

The majority (81%) of respondents who were prescribers agreed there was a connection between their prescribing and the emergence and spread of antibiotic-resistant bacteria, yet only 64% felt they have a key role in controlling antibiotic resistance (Supplementary Figure S4).

Across the devolved administrations, the percentage of survey participants agreeing with the motivation statements varied, ranging from 79% in Wales to 88% in Northern Ireland (Supplementary Figure S5).

### 3.6. Behaviour (Giving out Resources or Advice) and Barriers

In total, 59% of UK respondents had prescribed, administered or dispensed antibiotics at least once in the previous week (Supplementary Table S9), while almost 14% of the UK participants had not prescribed, administered or dispensed any antibiotics.

Of the prescribers, 21% had given out resources (e.g., leaflets or pamphlets) and 61% had given out advice on prudent antibiotic use at least once in the previous week to participating in the survey (Supplementary Figure S6).

The most common reasons respondents (*n* = 1671) gave for not providing resources or advice as frequently as they prescribed, administered or dispensed antibiotics were lack of resources (19%), insufficient time (11%) and the patient being uninterested in the information (7%) (Supplementary Table S10). Only 8% of HCWs stated they were able to give out advice or resources as needed.

### 3.7. Awareness of National Initiatives and Campaigns, and Perceived Effectiveness

Almost two-thirds (63%) of survey participants agreed that there has been good promotion of prudent antibiotic use and antibiotic resistance in the UK (Supplementary Figure S7). The results varied among HCWs across the devolved administrations (Supplementary Figure S8), with the most respondents agreeing from Northern Ireland (76%) and the least from Wales (54%).

**Figure 3:**
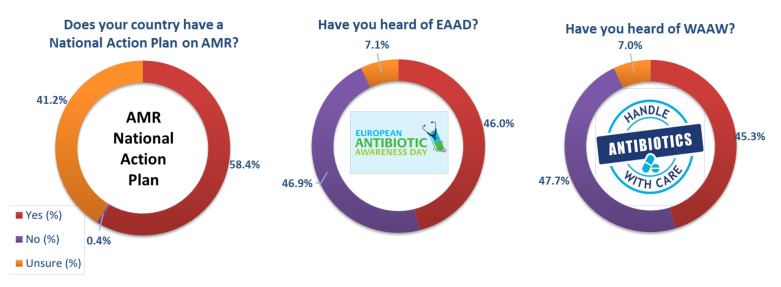
Awareness of campaigns in the UK; NAP (*n* = 2060), EAAD (*n* = 2070) and WAAW (*n* = 2054).

Fifty-eight percent of UK respondents were aware of the National Action Plan (Figure 3). Less than half of respondents were aware of either EAAD (46% aware) or WAAW (45%), and approximately 30% felt that national campaigns had been effective. Respondents in the UK were mostly undecided (55%) on the campaign’s effectiveness. Comparative data for the EU/EEA is available in Supplementary Figure S9. Focusing in on the UK constituent countries, participants from England agreed the most that national campaigns are effective (32%), whereas participants from Northern Ireland disagreed the most (24%) (Supplementary Figure S10).

**Figure 4:**
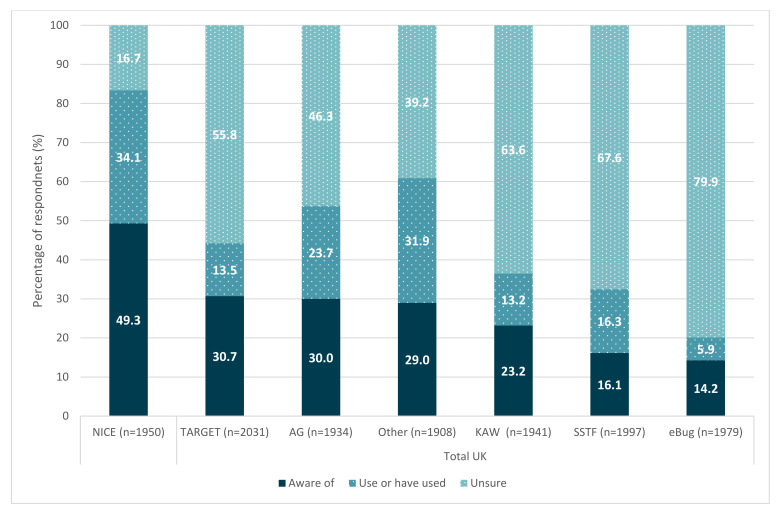
Awareness of campaigns, projects and platforms related to antibiotic use and AMR in the UK.

Across the UK as a whole, the most commonly known resources were the National Institute for Health and Care Excellence (NICE) guidelines (49%), Treat Antibiotics Responsibly, Guidance, Education and Tools (TARGET) (31%) and Antibiotic Guardian (30%) (Figure 4). The most commonly used resources were the NICE guidelines (34%), resources other than those listed (32%) and Antibiotic Guardian (AG) (24%). Across the devolved nations results varied; however, NICE guidelines were the most commonly known and used resource across the four countries (Supplementary Figure S11).

In total, 49% percent of respondents stated they were Antibiotic Guardians; however, only 21% could recall their pledge (Supplementary Figure S12). Results varied across the four nations; England had the highest percentage of Guardians (56%) and Scotland the lowest (31%) (Supplementary Figure S12). Over three-quarters (76%) of UK HCW respondents had seen or heard the KAW advert on television (TV) or radio (Supplementary Figure S13).

Less than a third (29%) of UK respondents stated they were prescribers of antibiotics, the majority of which were medical doctors (46%), then nurses (29%) and pharmacists (19%) (Supplementary Table S11). The majority of prescribers agreed they considered antibiotic resistance when treating a patient (92%), they have a key role to play in helping control antibiotic resistance (88%) and they are confident when making antibiotic-prescribing decisions (90%) (Table 2). Almost all prescribers were confident in the antibiotic guidelines available (96%), and they have easy access to those guidelines (94%).

**Figure 5:**
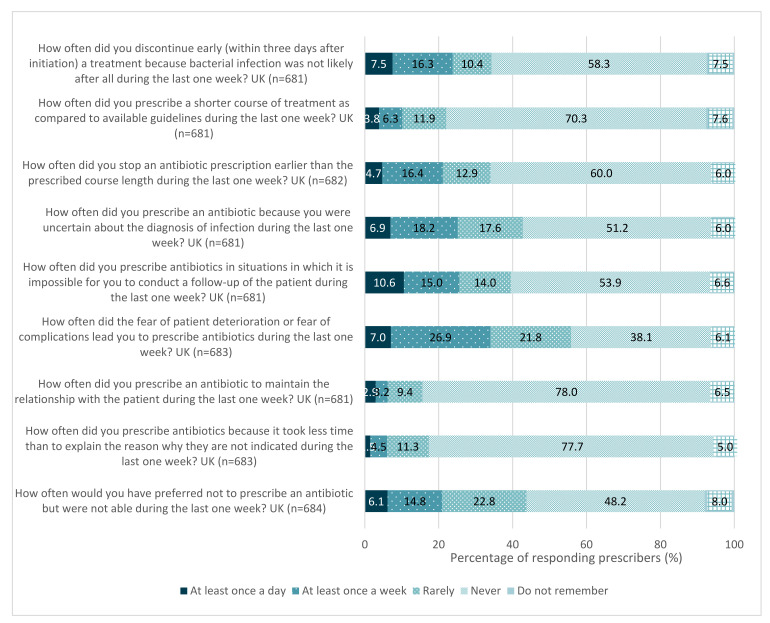
Reasons prescribers initiated antibiotic prescriptions when they would have preferred not to in the previous week, UK, 2019.

Almost three-quarters (74%) of respondents agreed they feel supported not to prescribe antibiotics when they are not necessary. Over 20% of prescribers prescribed an antibiotic at least once in the past week when they would have preferred not to (Figure 5). The reasons given for this were the fear of patient deterioration or complications (34%) and uncertainty in their diagnosis (25%).

### 3.8. Summary Recommendations for Action from Stakeholder Workshop

- Further analysis of the data should be undertaken to compare differences across professional groups and differences across settings.
- Sepsis messaging should be more strongly linked to antimicrobial resistance and focus on preventative measures.
- Facebook presents an opportunity to engage with UK healthcare workers. Campaigns, initiatives and interventions should be promoted on both Twitter and Facebook.
- Key organisations should be encouraged to produce a short video for WAAW outlining their AMR and AMS activities and available resources.
- All recommendations should align with the COM-B behaviour change wheel.

## 4. Discussion

This is the first UK-wide study assessing multidisciplinary HCWs’ knowledge, attitudes and behaviour towards antibiotics, antibiotic use and resistance. As a sub-study of the multicountry EU/EEA survey, the UK results present the most comprehensive and detailed dataset for the UK so far, with responses from 2404 participants across a variety of age groups, professions and prescribing settings. All data were collected prior to the COVID-19 pandemic, providing insight into a population’s baseline level prior to the widespread communication of prescribing measures that occurred throughout the pandemic. This analysis gives evidence for developing interventions targeted at HCWs, informing policy making, improving the reach and impact of public awareness campaigns and supporting HCWs with their antimicrobial stewardship.

The study exceeded its calculated quota sample by 74%, significantly increasing the generalisability and validity of the results at a country level. The UK constituted the largest proportion of respondents across the EU, equating to 13% of the total participants. However, it is worth noting that the majority of UK responses were from participants in England (50%) and Scotland (40%), limiting the direct application of these results to Northern Ireland and Wales and biasing the results to be more representative of Scotland in the context of the UK population. Volunteer bias risk is high in surveys; it is likely that those HCWs most interested in AMR completed the survey. Equally, it is possible that the wider prescribing population may not have the same level of interest and awareness of AMR. The authors also acknowledge the survey did not include the assessment of all elements of antimicrobial stewardship HCWs may utilise. Additional clinical decision and support tools, such as Electronic Health Records, and quality improvement initiatives, such as auditing, are valuable AMS strategies.

A key strength is the representation across all healthcare settings. In addition, the quota sample size was exceeded for key healthcare professional groups who prescribe, administer or dispense antibiotics such as physicians, nurses and pharmacists. [21]. In the UK, the largest groups of survey respondents were nurses and pharmacists, these two professions made up the highest percentage of the target sample size, at 175% and 462%, respectively (Supplementary Table S2). This emphasises the need to target future interventions and communications to these engaged groups and reinforces that they, along with physicians, can contribute to positive change in combating AMR. This differed to the EU/EEA in which physicians made up the greatest proportion of respondents, followed by nurses and pharmacists [16]. This is to be expected, as in mainland Europe, only medical doctors are able to prescribe.

The findings highlighted gaps in knowledge across all healthcare professional groups. Although HCWs understood the risk of side effects to the patient, fewer understood that antibiotic use increases a person’s risk of AMR. Similarly, despite over 90% of HCWs understanding that healthy people can be colonised with antibiotic-resistant bacteria, one in five UK HCWs knew resistant bacteria can spread between people. It is important that prescribers understand the wider implications of AMR on patient outcomes. The positive results in the knowledge test correlate with earlier studies; in the United States, Hamilton et al. found that 97% (*n* = 194) of nurse practitioners understood antibiotics can harm the patient and inappropriate use can cause resistance (97%) [11].

Overall, EU/EEA knowledge was stronger than the UK’s regarding spread, but marginally weaker when it came to understanding a patient’s increased risk of AMR. Future campaigns should consider including messages on the transmission of AMR, addressing the gap in knowledge identified through this study. National and local educational groups could also include these messages within their infection-related training materials.

Access to guidelines for managing infections was high in the UK (78%); however, fewer HCWs had access to the materials needed to provide advice and opportunities to provide that advice (68% and 62%, respectively). Improving opportunities to provide advice is a particular area of concern in the UK, as fewer UK respondents agreed with this statement in comparison to the EU/EEA as a whole. Increasing access to resources, as well as improving opportunities for HCWs to provide advice to patients on beneficial and judicious antibiotic use, are key to maximising HCWs’ antimicrobial stewardship (AMS) capabilities. Intervention types should align with the behaviour change wheel [18] and focus on training, environmental restructuring, modelling exemplar behaviours and enablement [22] to produce durable development and improvement.

All HCWs can contribute to tackling AMR via good infection prevention and control practices; however, only 63.9% of respondents felt they had a key role in controlling AMR. As would be expected, the equivalent figure was higher in prescribers, where 88.4% felt they had a key role. Further engagement with HCWs should focus on empowering them in optimal AMS prescribing practices and reinforcing their vital role in tackling resistance. Educating HCWs on the wider context of One Health actions and impact may encourage their ensuing motivation for behaviour change, highlighting the importance prescribing behaviour can have on this global health challenge [23].

Findings from this study provide insight into the reasons why practitioners do not give AMS advice and are reinforced by the EU/EEA survey results. A lack of resources is the most frequently cited barrier (19% of respondents) to giving out advice. This can be addressed by implementing patient educational resources about antibiotics and resistance and making them easily accessible for practitioners. Resources should be accompanied by national and local campaigns to increase awareness and outline their purpose for use. These findings provide evidence of the need to further promotion and dissemination of available AMS resources such as TARGET (Treat Antibiotics Responsibly, Guidance, Education and Tools), EAAD/WAAW resources, When Should I Worry’ patient brochure or ‘Treating Your Infection’ patient leaflet. More than half of respondents answered ‘unsure’ when asked if they were aware of or have used the TARGET resources; in a small evaluation of TARGET resources, participants (including HCWs) have reported these resources are useful, reinforcing the value of further dissemination [24]. A limitation of this study is that it is not known whether participants who had access to the resources were more likely to provide advice on antibiotics. It is also pertinent to understand barriers and enablers to their use. Insufficient time was cited as the second most common barrier to providing advice across both the UK and EU/EEA. This is concordant with the UK finding that only 62% of HCWs felt they had sufficient opportunity to provide advice on antibiotics (Table 1).

It is important to consider all barriers associated with prudent prescribing, taking into account the professional group, and investigate them further in order to best support HCWs to overcome these challenges. The fear of patient deterioration or complications was the foremost reason (34% of respondents), both in primary and secondary care settings, for issuing an antibiotic prescription, and uncertainty in diagnosis was the second most common reason (25%). These findings correlate with the literature; fear has been cited as a common barrier to tackling AMR amongst community healthcare providers and physicians, alongside patient pressure, time and systemic overworking [12,13,25,26].

Other factors to consider include consultation time constraints, prescriber–patient relationships and patient awareness and expectations in receiving antibiotic prescriptions. Further qualitative research is warranted to improve our understanding of these factors and implement useful and successful interventions.

International and national campaigns provide means through which to reach more population numbers. WAAW and EAAD managed to reach nearly half of the UK respondents, with 45% stating they were aware of WAAW, and 46% were aware of EAAD. Nationally, 76% of UK HCWs had seen or heard the KAW advert on TV or radio. Conversely, in answer to a separate question, only 36% of respondents stated they were aware of or had used KAW materials.

Tackling AMR requires engagement at individual, population and government levels. The Antibiotic Guardian (AG) initiative, of which more than half of the study cohort (53.7%) are aware of or have used, includes the proactive engagement of ‘pledging’ [26]. The evaluation of the AG campaign demonstrated an increased commitment of healthcare workers, scientists/educators and the public in tackling AMR, increased self-reported knowledge and changed self-reported behaviour, particularly among people with prior AMR awareness [27,28]. Pledging has been identified as an effective and inexpensive driver for behaviour change relating to antibiotic use [27]. It provides individuals with concrete actions and examples of how to tackle AMR and at the same time includes them in the collective movement to drive change.

The survey identified that Facebook was the most popular social media platform used by UK HCW respondents for professional use. This communication channel presents an opportunity to disseminate information and engage with HCWs. A significant proportion (44%) of participants did not use any of the social media platforms listed. In future surveys, it would be worth expanding communication efforts beyond social media channels to engage with participants in a more inclusive manner, ultimately increasing impact.

It is important that the UK considers the findings of this study and advocates for next steps and actions to tackle the public health threat of AMR. This study provides baseline data for policy makers on AMR awareness and AMS involvement within the HCW population. It would be insightful to undertake the survey in the wake of the COVID-19 pandemic, to help assess what, if any the impact pandemic may have had (with the consistent infection prevention and control measure messages), on HCW populations and their awareness of AMR and AMS. Moreover, conducting regular surveys to evaluate ongoing initiatives would be valuable in order to measure impacts and identify areas for improvement.

The implementation of actions at both national and local levels is imperative to changing practice and behaviour. The effectiveness of interventions on improving antibiotic prescribing depends on prescribing behaviours and on perceived and actual barriers to change. Multifaceted educational interventions occurring on multiple levels will only be effective after local barriers to change are addressed [29,30,31]. Educational, training or communications materials for HCWs in the UK should consider the findings of this study when developing content/curriculum and embed behaviour change strategies as part of developing interventions.

## 5. Conclusions

These findings strengthen our understanding of UK HCWs’ knowledge, attitudes and behaviours towards antibiotics and antibiotic resistance and provide an evidence base for guiding future AMS activities, interventions and research priorities. The results highlight HCWs’ comprehension of AMR; however, specific knowledge gaps across professional groups should be targeted in future educational initiatives. Training materials should address the risk of AMR spread and the impact this has for individuals and the public. Resources, initiatives and future campaigns should be promoted across a variety of social media platforms to maximise engagement with UK HCWs. Further evaluation is needed to identify the most effective method for communicating with practitioners that do not use social media professionally. The findings identify potential barriers faced by HCWs. Subsequent evaluation is needed of the barriers and enablers to inform future interventions on improving awareness of and access to antibiotic resources, increasing the opportunity for HCWs to use these resources and supporting prescribers not to prescribe when they feel antibiotics are not needed. Future surveys should be undertaken to longitudinally inform and evaluate strategies in this field. Research on novel interventions should embed behaviour change strategies, aligning with the behaviour change wheel, to produce effective, long-lasting and positive behaviours relating to antibiotics and their use.

## Figures and Tables

**Table 1:** Percentage of respondents who agreed or strongly agreed with the opportunity statements.

Statement	UK	EU/EEA
I have easy access to guidelines I need on managing infections (UK, *n* = 2291; EU/EEA, *n* = 14,301)	79.7%	75.1%
I have easy access to the materials I need to give advice on prudent antibiotic use and antibiotic resistance (UK, *n* = 2291; EU/EEA, *n* = 14,299)	67.5%	67.5%
I have good opportunities to provide advice on prudent antibiotic use to individuals (UK, *n* = 2291; EU/EEA, *n* = 14,296)	61.6%	72.3%

**Table 2:** Responses from UK prescribers to the motivation statements.

Motivation Statement	Agree or Strongly Agree	Disagree or Strongly Disagree	Undecided	Do Not Understand the Question
I am confident making antibiotic prescribing decisions (*n* = 682)	90.0	3.2	6.6	0.1
I have confidence in the antibiotic guidelines available to me (*n* = 681)	95.6	1.5	2.8	0.1
I have a key role in helping control antibiotic resistance (*n* = 681)	88.4	3.7	7.6	0.3
I consider antibiotic resistance when treating a patient (*n* = 681)	91.6	3.1	5.0	0.3
I have easy access to antibiotic guidelines I need to treat infections (*n* = 681)	94.0	1.9	4.0	0.1
I feel supported to not prescribe antibiotic when they are not necessary (*n* = 680)	74.3	8.4	17.1	0.3

## Data Availability

Additional data from the study can be found via https://www.ecdc.europa.eu/en/publications-data/survey-healthcare-workers-knowledge-attitudes-and-behaviours-antibiotics (accessed on 1 April 2021).

## Supplementary Materials

The following supporting information can be downloaded at: https://www.mdpi.com/article/10.3390/antibiotics11081133/s1, Supplementary Material. Table S1: UK responses across the devolved administrations; Figure S1: Participation in the survey by region in England; Table S2: UK quota sample size and responses to the survey; Table S3: Percentage of UK respondents by profession; Table S4 Respondents’ age, gender and professional setting; Table S5: Percentage of UK and EU/EEA respondents that use the different social media platforms; Figure S2: The percentage of respondents answering all seven knowledge test questions correctly - by profession; Figure S3a: Percentage of respondents answering question 5 correctly, by professional group; Figure S3b: Percentage of respondents answering question 6 correctly, by professional group; Table S6: Respondents answering all seven knowledge test questions correctly, by UK constituent; Table S7: Respondents answering the One Health knowledge question correctly; Table S8: Respondents answering the Hand Hygiene knowledge question correctly; Figure S4: Percentage of respondents who agree with the following motivation statements; Figure S5: Percentage of UK respondents agreeing to the motivation statements (by constituent); Table S9: The frequency of prescribing/administering/dispensing antibiotics during the last one week; Figure S6: Percentage of respondents that gave out resources or advice; Table S10: Barriers: Most common reasons why UK healthcare workers were unable to provide advice or resources to their patients in a one-week period; Figure S7: Percentage of respondents who agree with the campaign impact and effectiveness statements in the UK and EU/EEA; Figure S8: Percentage of respondents who agree with the campaign impact and effectiveness statements across the devolved administrations; Figure S9: Awareness of campaigns in a. the UK b. the EU/EEA; Figure S10: Perceived effectiveness of EAAD and WAAW in the UK; Figure S11: Awareness of campaigns, projects and platforms related to antibiotic use and AMR in the UK; Figure S12: Engagement with Antibiotic Guardian; Figure S13: Awareness of the Keep Antibiotics Working Campaign; Table S11: Professions of prescribers in the UK; Table S12: Setting of prescribers in the UK; Figure S14: Responses from UK prescribers to the motivation statements.
