# Supplementary material for: Knowledge and Attitudes about Antibiotics and Antibiotic Resistance of 2404 UK Healthcare Workers
Source: Antibiotics (Basel). 2022 Aug 21;11(8):1133. doi: 10.3390/antibiotics11081133 (PMC9404832; doi:10.3390/antibiotics11081133)

antibiotics MDPI

Table S1. UK responses across the devolved administrations

Country	Number of responses	
	n	%
England	1,210	50.73
Northern Ireland	51	2.14
Scotland	949	39.79
Wales	175	7.34

Figure S1. Participation in the survey by region in England

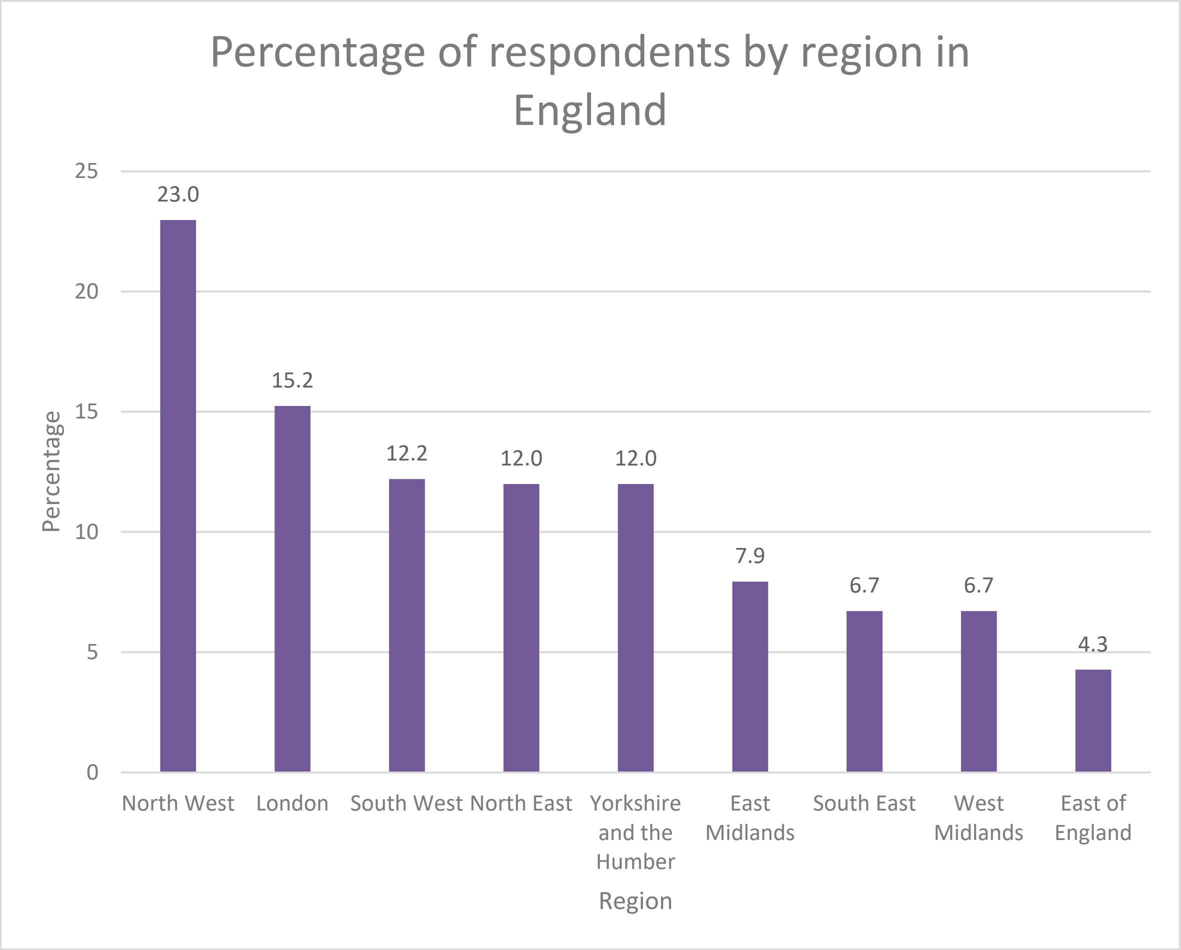

UK Region	Number of responses	
	n	%
East Midlands	39	7.93
East of England	21	4.27
London	75	15.24
North East	59	11.99
North West	113	22.97

South East	33	6.71
South West	60	12.2
West Midlands	33	6.71
Yorkshire and the Humber	59	11.99

Table S2. UK quota sample size and responses to the survey

	Target sample size	Actual number of responses	% of target sample size
Nursing Professionals (Nurses, nurse assistants & midwives)	548	962	175
Pharmacists	113	523	462
Physicians	365	427	117
Other healthcare workers	219	360	164
Dentists	70	22	32
All healthcare workers	1315	2294	174

Table S3. Percentage of UK respondents by profession

Professional Group	Number of responses	
	n	%
Nurse	892	37.1
Pharmacist	523	21.8
Medical doctor	427	17.8
Pharmacy Technician	153	6.4
Allied Health Professional	147	6.1
Scientist	68	2.8
Other healthcare worker	59	2.5
Nursing associate/assistant	47	2.0
Other	37	1.5
Midwife	23	1.0
Dentist	22	0.9
Unknown	5	0.2
Dental care professional	1	0.0

Table S4. Respondents' age, gender and professional setting (n = 2,404)

	Number of respondents	
**Gender identified with**	**n**	**%**
Female	1863	77%
Male	510	21%
prefer not to say	31	1%
	**Number of respondents**	
**Age (years)**	**n**	**%**
<18	1	0%
>66	21	1%
18-25	97	4%
26-35	463	19%
36-45	619	26%
46-55	809	34%
56-65	376	16%
prefer not to say	18	1%
**Predominant practice**	**Number of respondents**	
**setting**	**n**	**%**
Hospital	1,382	57.49
Community	752	31.28
Governmental organisation	72	3
Pharmacy	68	2.83
University	41	1.71
Public Health institute	31	1.29
LTCF	25	1.04
Other	13	0.54
Professional body	9	0.37
Not specified	3	0.12
Industry	1	0.04
Unknown	7	0.29

Table S5. Percentage of UK and EU/EEA respondents that use the different social media platforms

Social Media	Number of responses (n = 2,404)	
	**n**	**%**
None	1058	44
Facebook	577	24
Twitter	553	23
LinkedIn	240	10
Google +*	192	8

YouTube	144	6
Instagram	120	5

* *(likely confusion with google search engine)*

Figure S2. The percentage of respondents answering all seven knowledge test questions correctly - by profession (n=2,403)

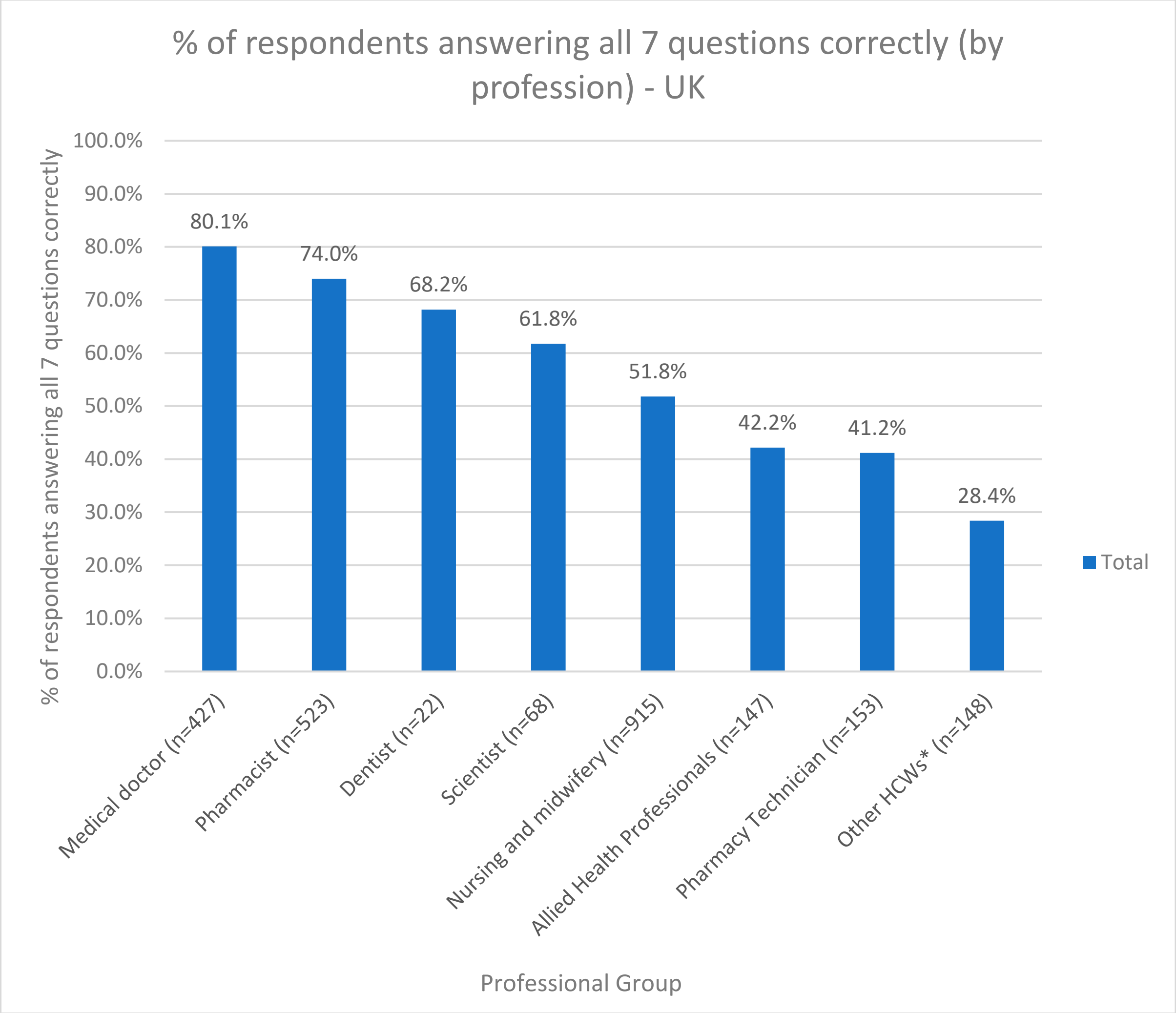

Prof Group	Number of respondents n	%
Medical doctor	342	80.1%
Pharmacist	387	74.0%
Dentist	15	68.2%
Scientist	42	61.8%
Nursing and midwifery	0	51.8%
Allied Health Professionlas	62	42.2%
Pharmacy Technician	63	41.2%
*Other HCWs (Nursing associates, dental care professionals, unknown, other)	42	28.4%

Figure S3a. Percentage of respondents answering question 5 correctly, by professional group

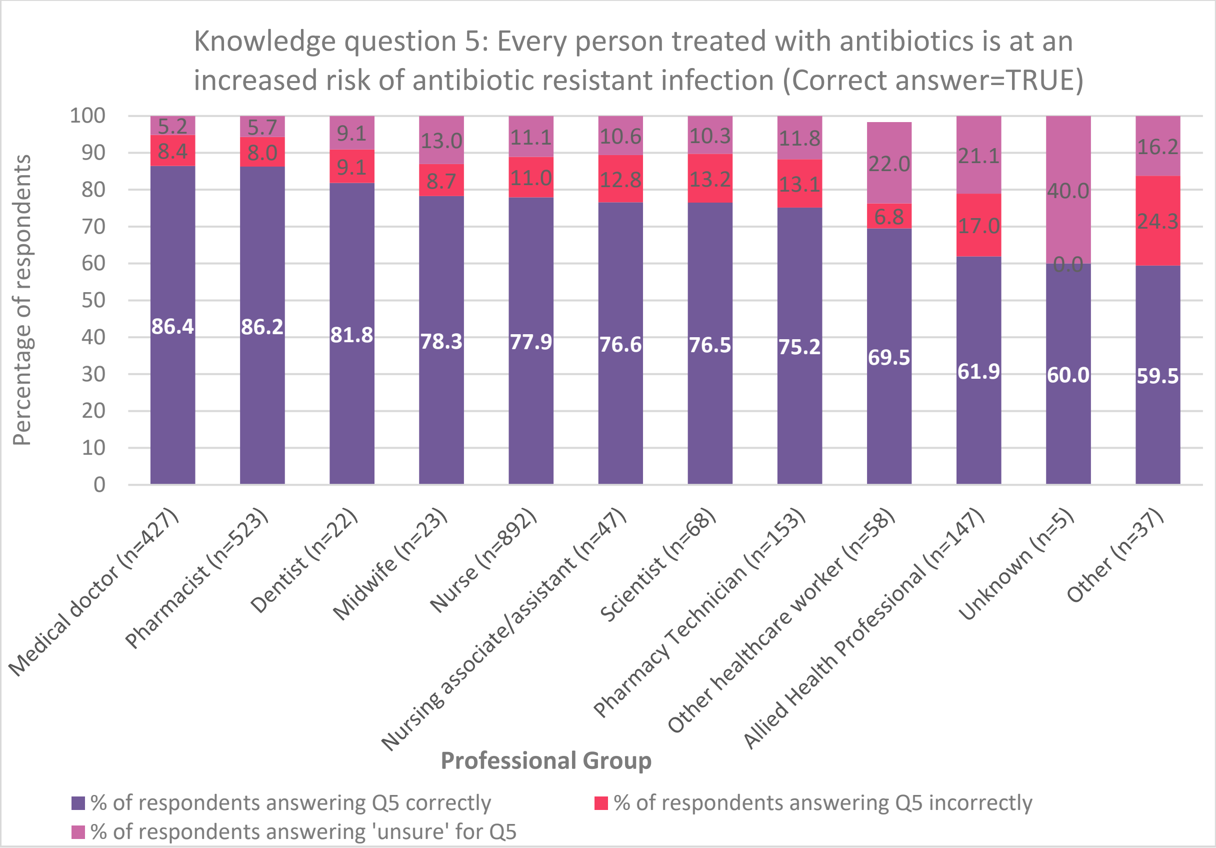

Figure S3b. Percentage of respondents answering question 6 correctly, by professional group

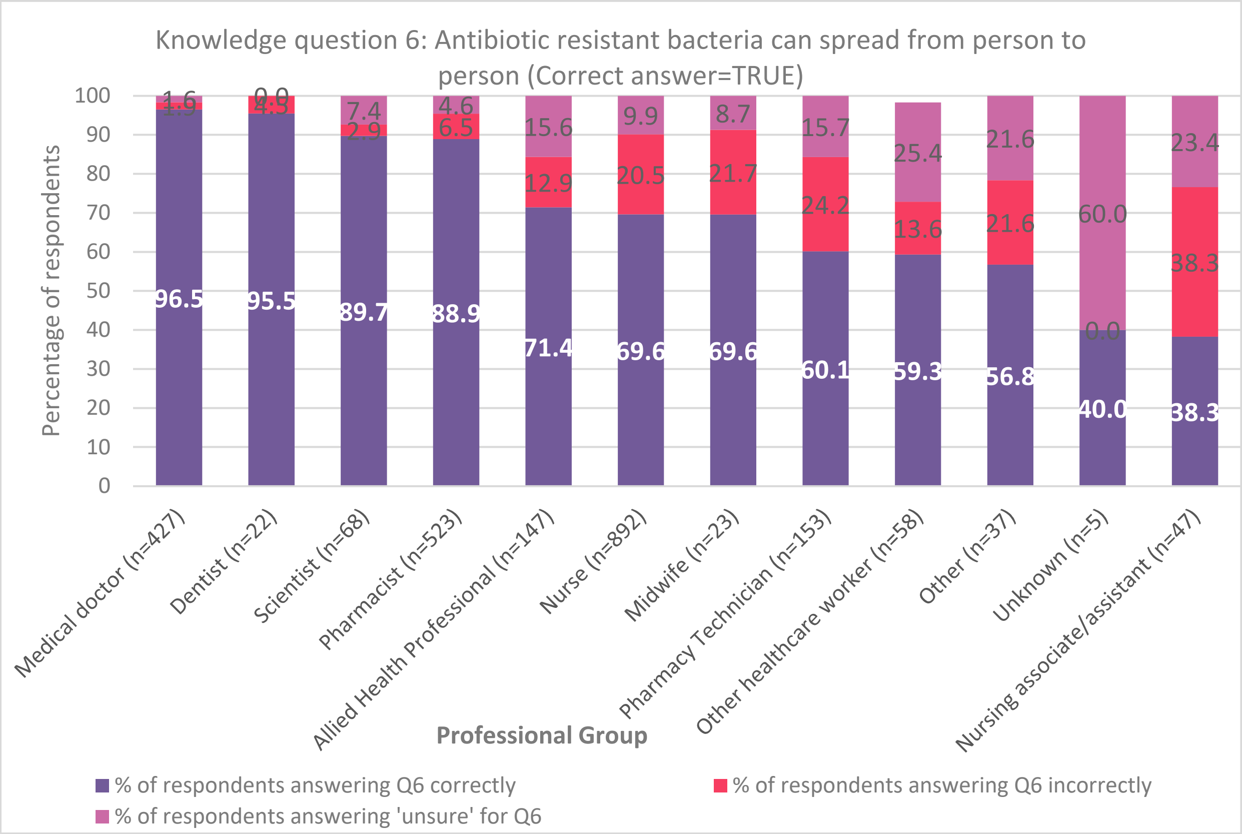

Table S6. Respondents answering all seven knowledge test questions correctly, by UK constituent

UK Constituent	Number of respondents answering all 7 questions correctly	% respondents answering all 7 questions correctly
Northern Ireland (n=51)	41	80.4%
England (n=1,209)	755	62.4%
Scotland (n=949)	532	56.1%
Wales (n=175)	95	54.3%
Total UK (n=2,384)	1427	59.4%
EU/EEA (n=18,348)	10,642	58.0%

Figure S4. Percentage of respondents who agree with the following motivation statements.

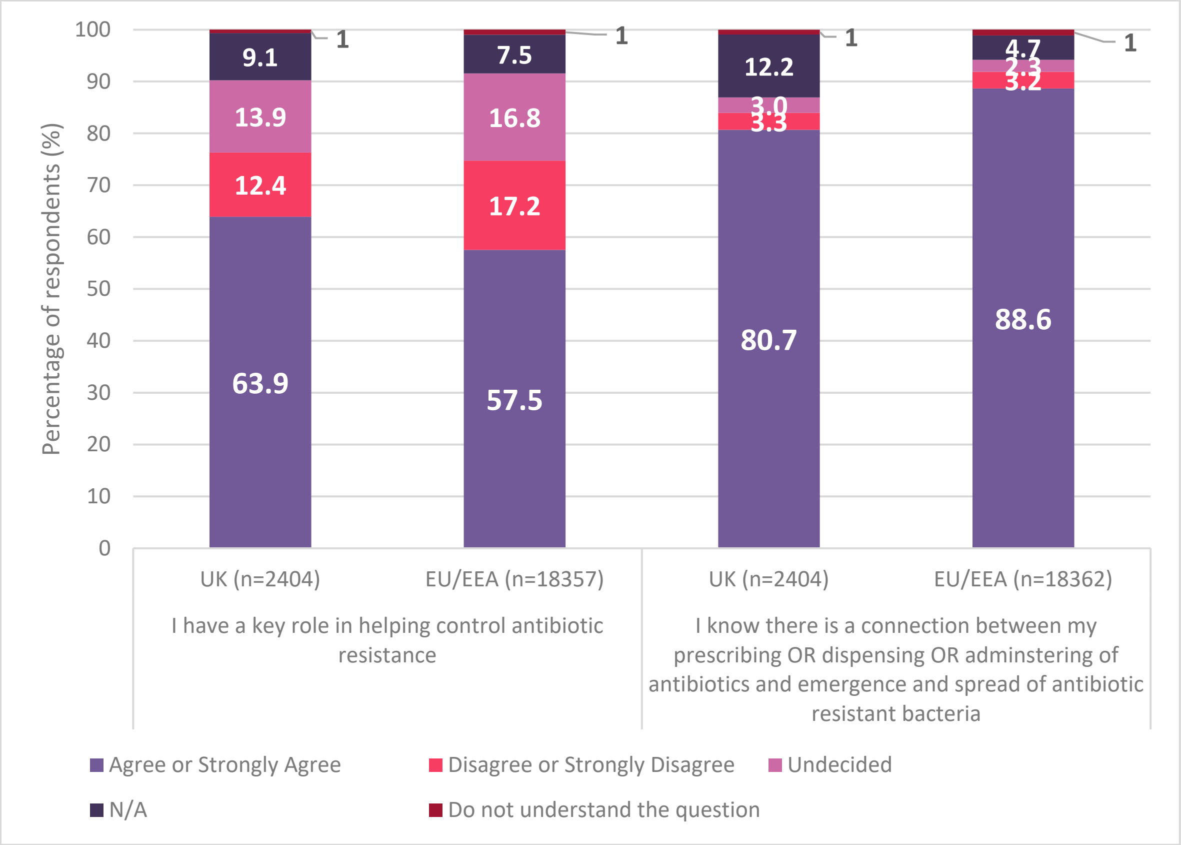

Figure S5. Percentage of UK respondents agreeing to the motivation statements (by constituent).

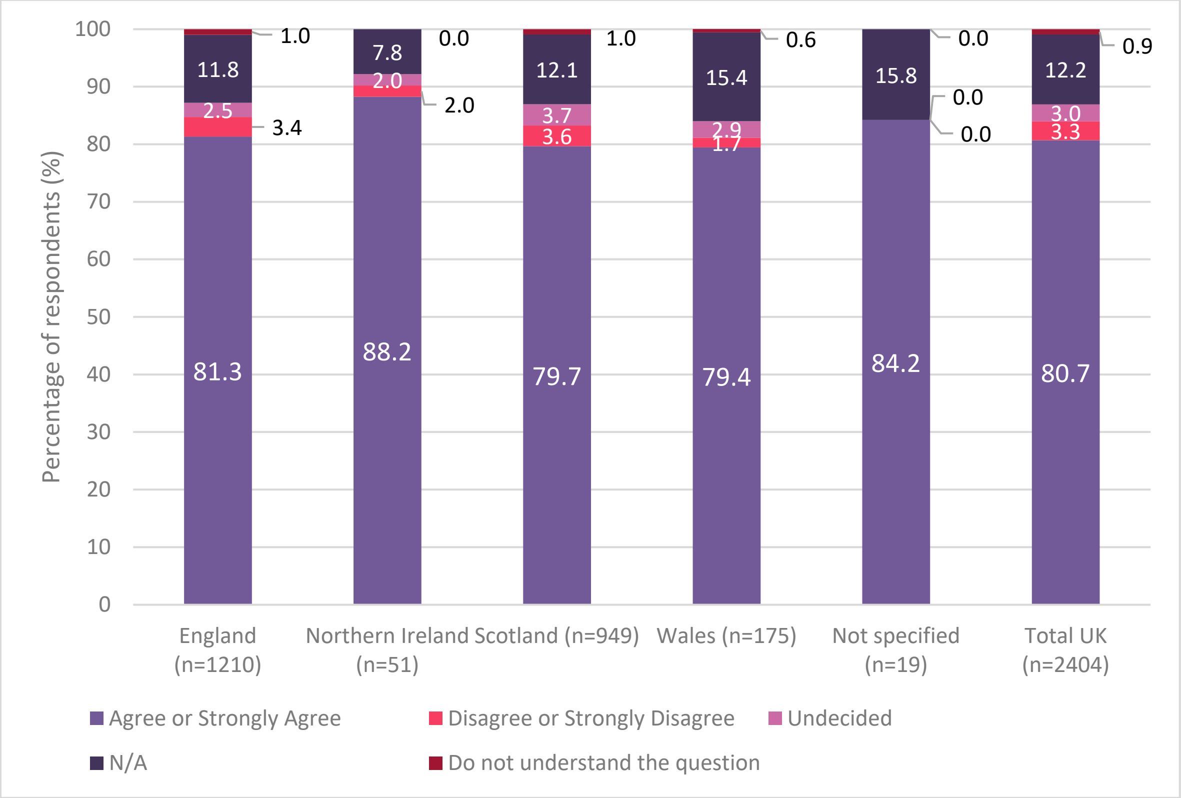

Table S7. The frequency of prescribing/administering/dispensing antibiotics during the last one week.

	At least once a week (%)	Rarely (%)	Never (%)	N/A (%)	Do not remember (%)
UK (n=1775)	59.4	6.1	13.9	20.1	0.6
EU/EEA (n=14294)	65.1	7.1	11.3	15.4	1.1

Figure S6. Percentage of respondents that gave out resources or advice

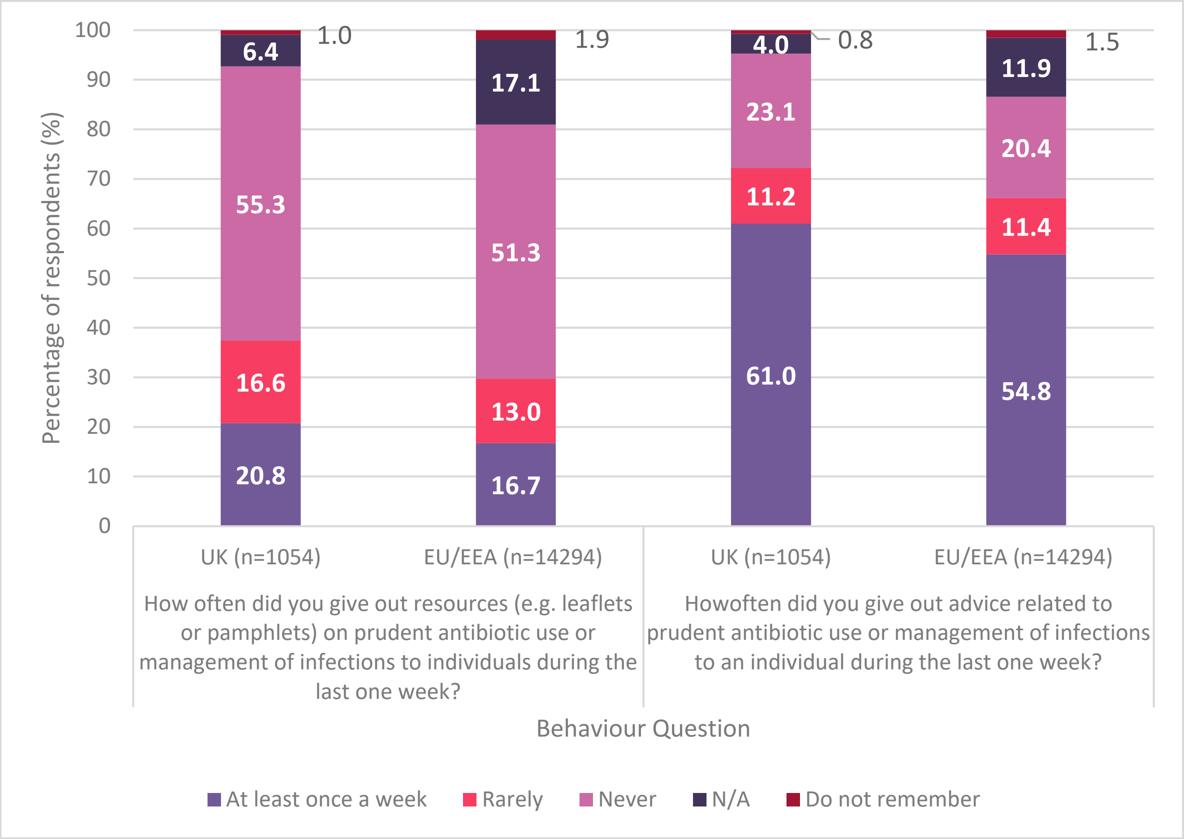

Table S8. Barriers: Most common reasons why UK healthcare workers were unable to provide advice or resources to their patients in a one-week period

Why were you not able to provide advice or resources as often as you prescribed OR administered OR dispensed antibiotics?	Frequency of chosen option (% of total selection) in the UK (n=1671)
Not applicable	43.6
No resources available	19.1
Insufficient time	11.3
Patient does not require information	8.6
I was able to give out advice or resources as needed	8.4
Patient uninterested in information	7.0
I was not sure what advice to provide	4.3
Difficulty getting patient to understand diagnosis	4.1
Language barriers	2.2

Figure S7. Percentage of respondents wo agree with the campaign impact and effectiveness statements in the UK and EU/EEA

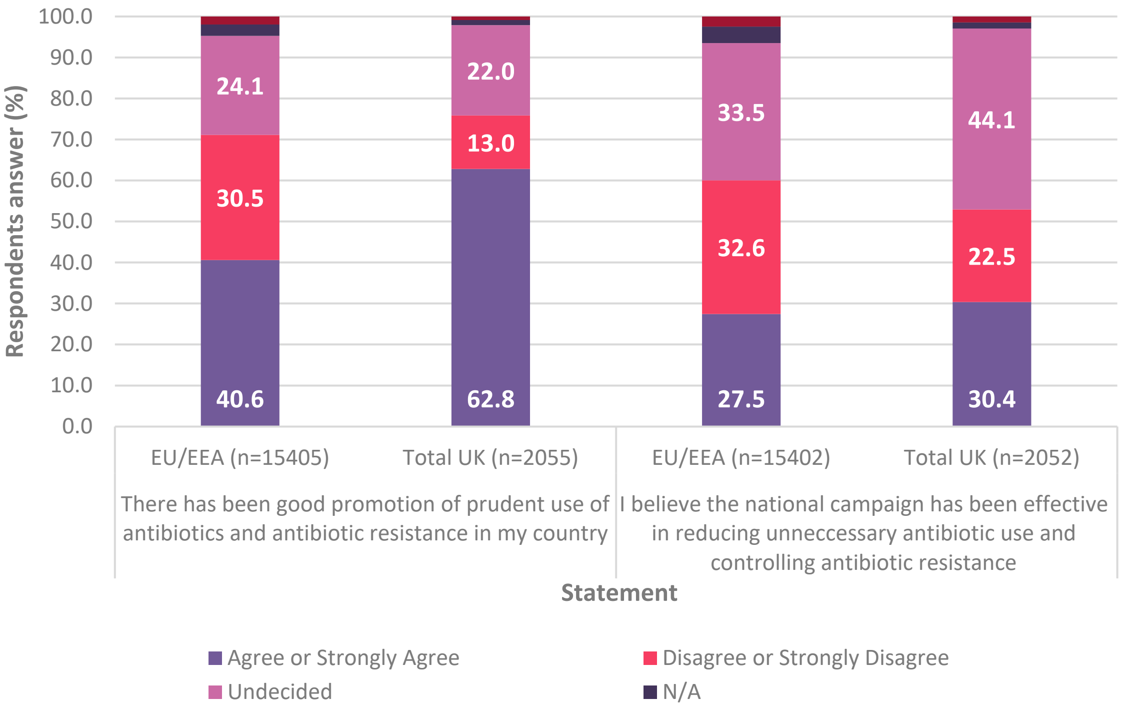

Figure S8. Percentage of respondents who agree with the campaign impact and effectiveness statements across the devolved administrations

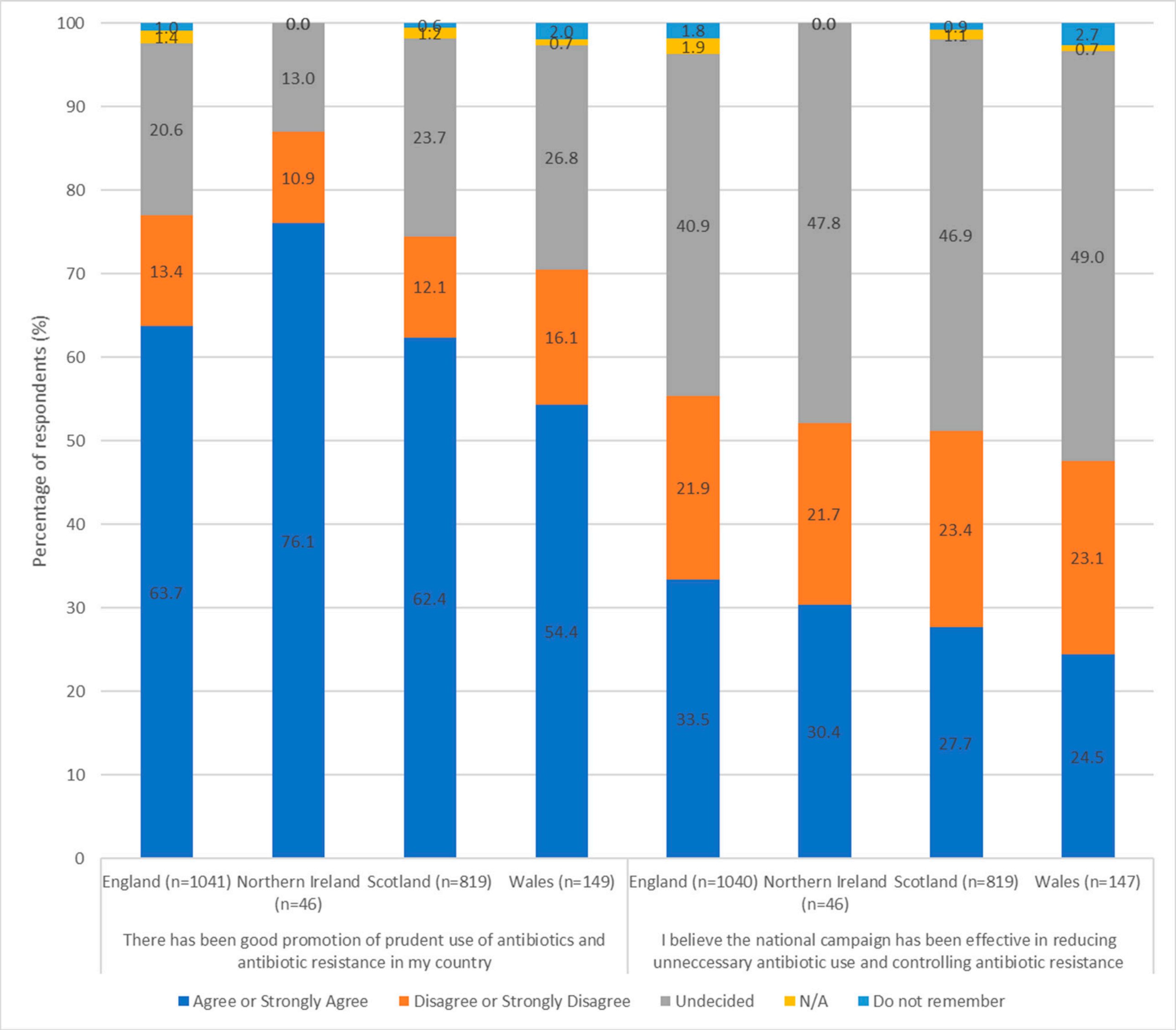

Figure S9. Awareness of campaigns in a. the UK b. the EU/EEA

a. **Awareness of NAP (n=2,060), EAAD (n=2070) and WAAW (n=2,054)**

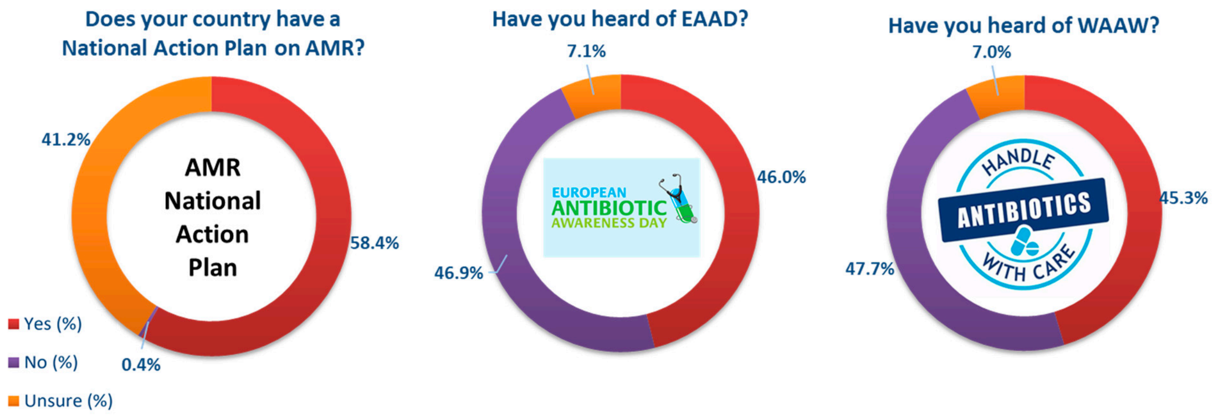

a. **b. Awareness of NAP (n=15,385), EAAD (n=15,518) and WAAW (n=15,397)**

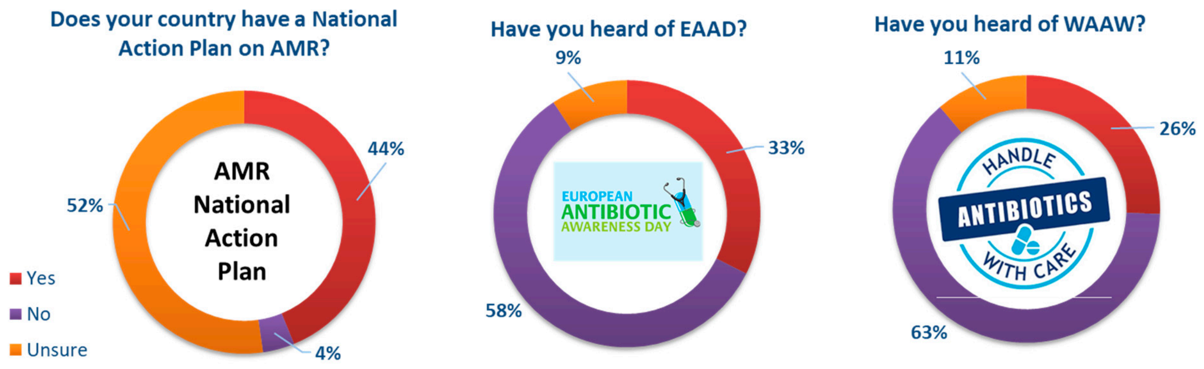

Figure S10. Perceived effectiveness of EAAD and WAAW in the UK

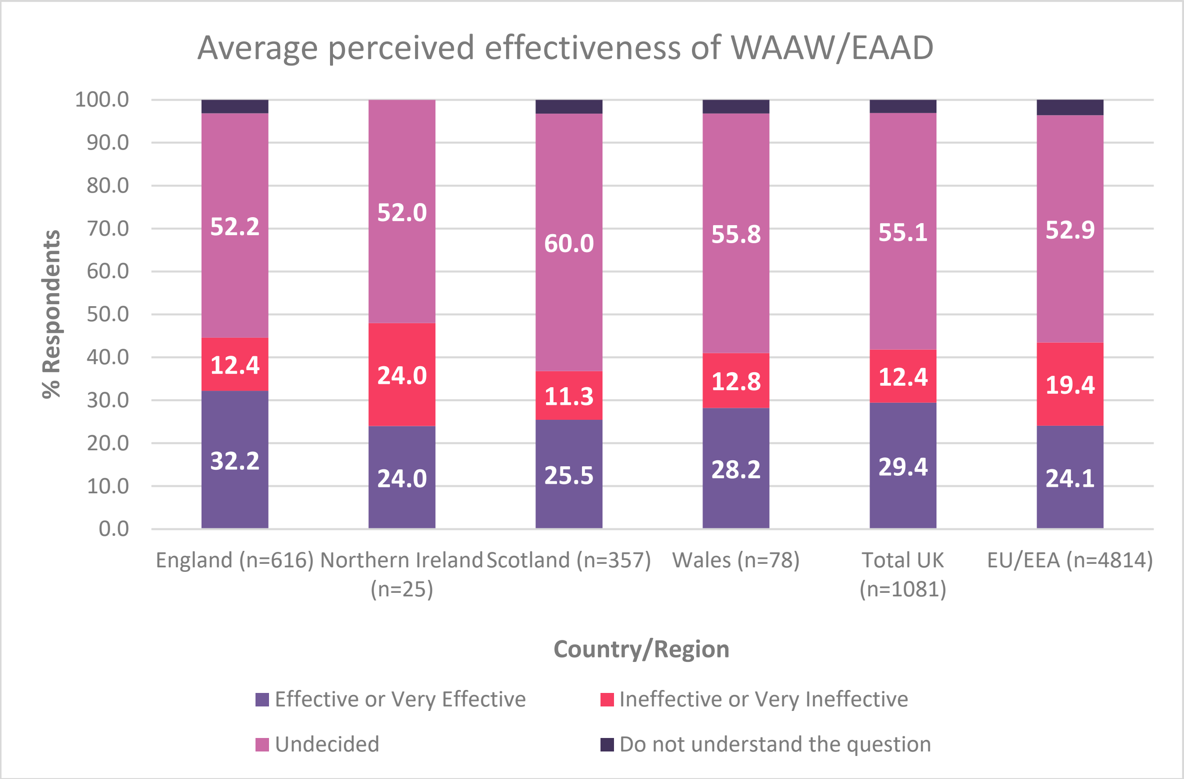

Figure S11. Awareness of campaigns, projects and platforms related to antibiotic use and AMR in the UK.

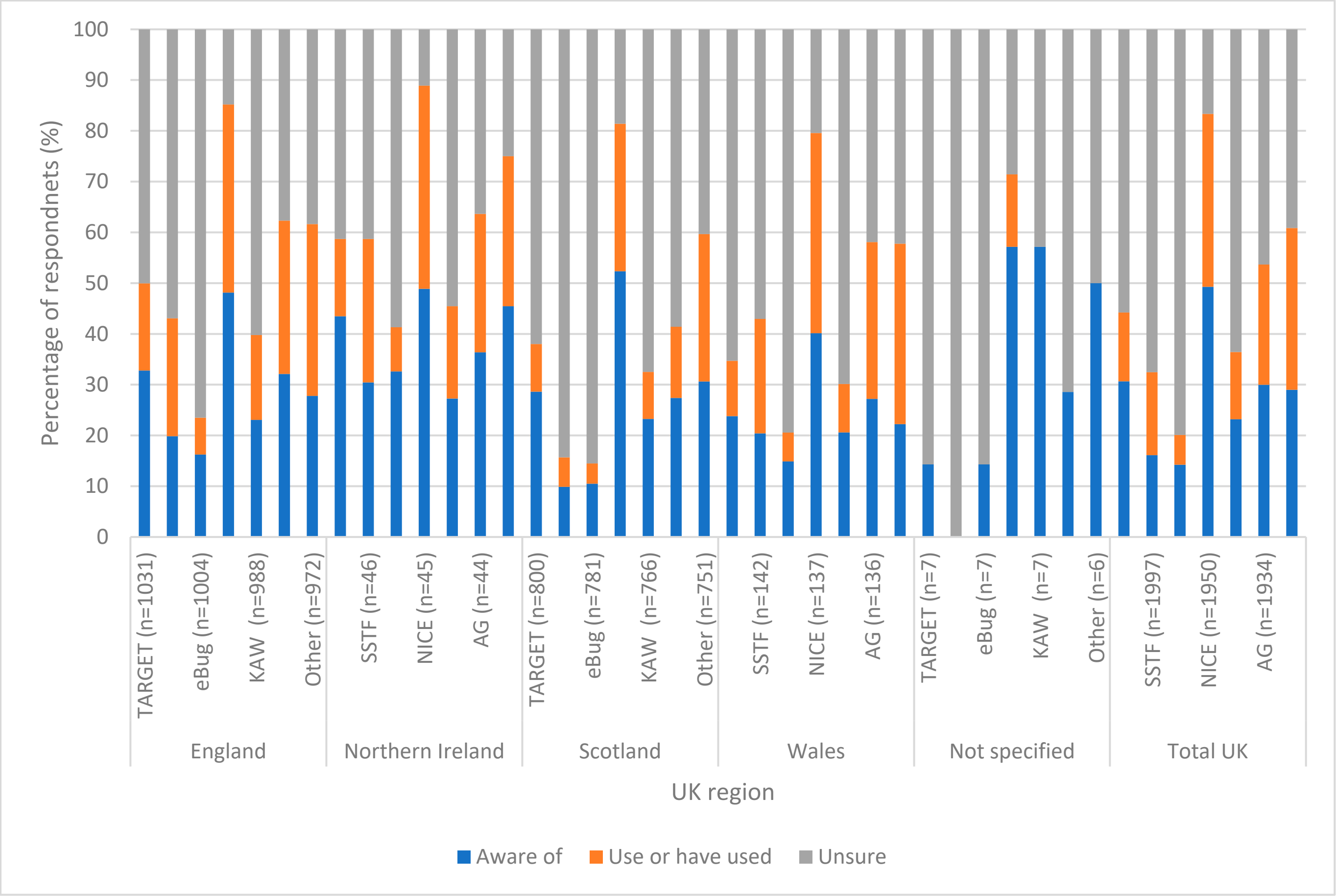

Figure S12. Engagement with Antibiotic Guardian

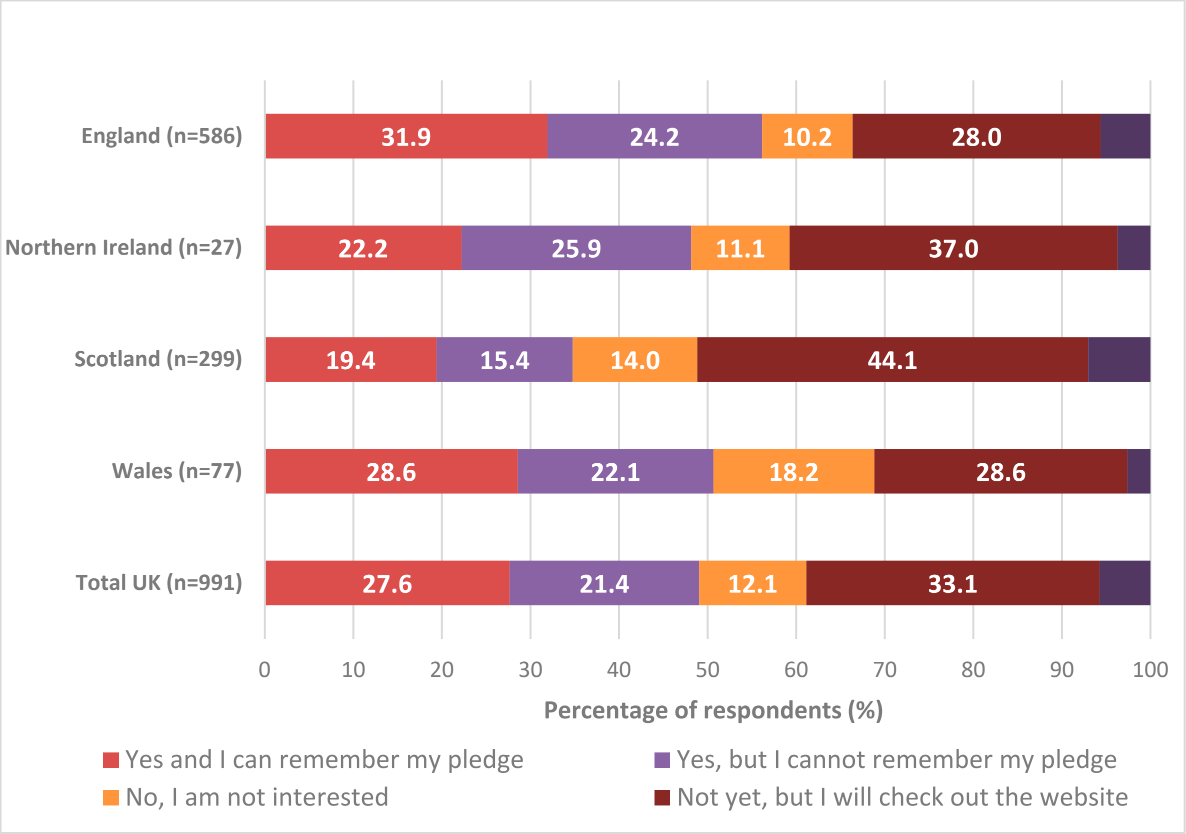

Figure S13. Awareness of the Keep Antibiotics Working Campaign

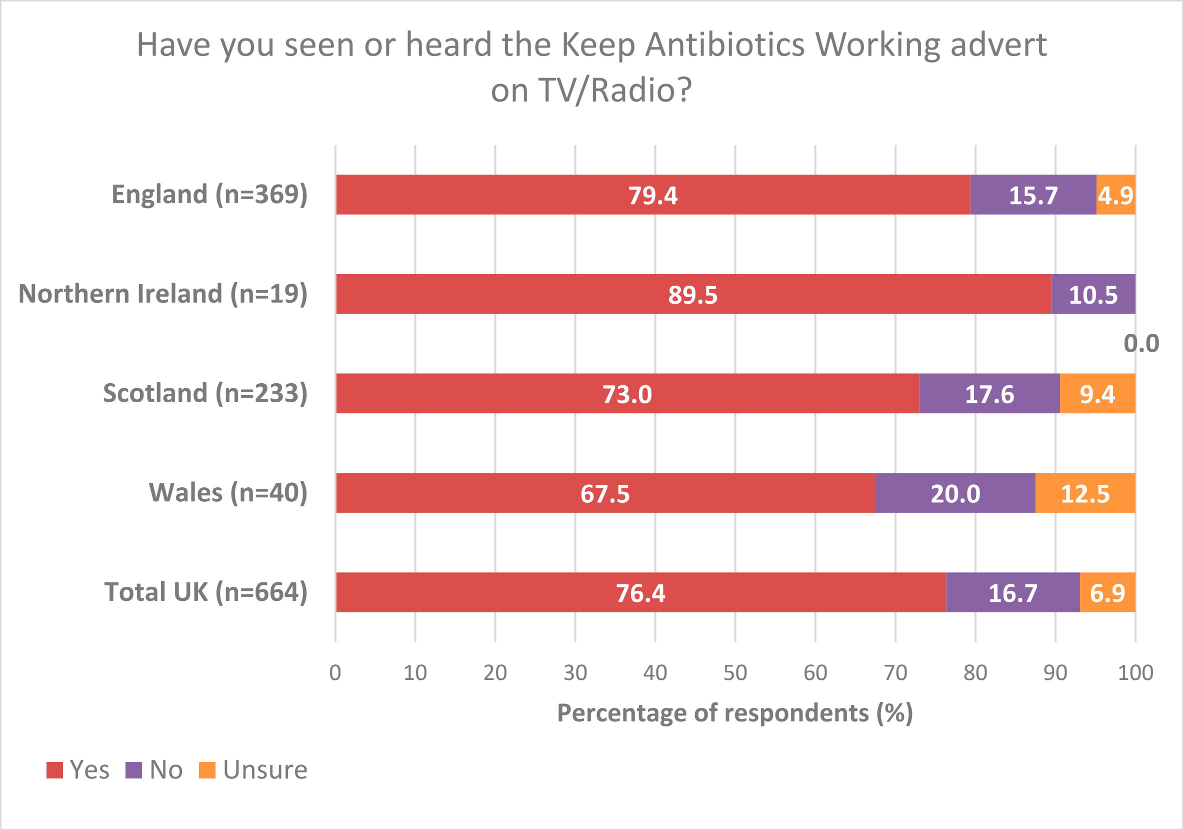

Table S9. Professions of prescribers in the UK

**Profession**	**Number of respondents that stated they were prescribers (%)** (n=699)
Medical doctor	318 (45.5)
Nurse	199 (28.5)
Pharmacist	131 (18.7)
Allied Health Professional	22 (3.1)
Dentist	17 (2.4)
Midwife	6 (0.9)
Other healthcare worker	2 (0.3)
Nursing associate/assistant	1 (0.1)
Other	1 (0.1)
Scientist	1 (0.1)
Unknown	1 (0.1)

Table S10. Setting of prescribers in the UK

Setting	Number of respondents that stated they were prescribers (%) (n=699)
Hospital	421 (60.2)
Community	246 (35.2)
Pharmacy	10 (1.4)
Governmental organisation	5 (0.7)
Long term care facility	5 (0.7)
Public health institute	3 (0.4)
University	3 (0.4)
Other	2 (0.3)
Unknown	2 (0.3)
Industry	1 (0.1)
Professional body	1 (0.1)

Figure S14. Responses from UK prescribers to the motivation statements

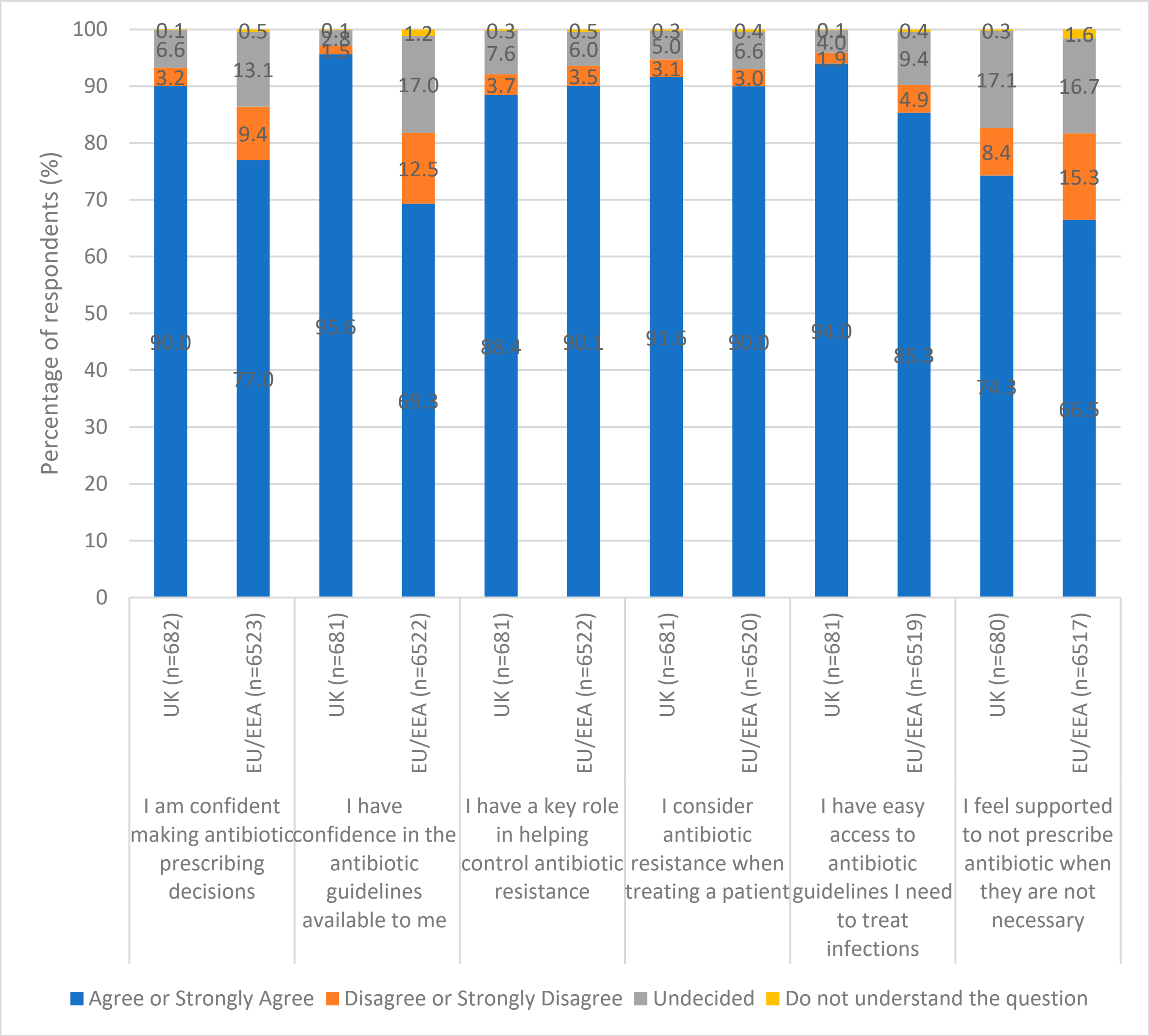

Supplement: Supplementary file 1 [file antibiotics-11-01133-s001.zip › antibiotics-1820274-suppl-done.pdf]
