# Supplementary material for: Knowledge and Attitudes about Antibiotics and Antibiotic Resistance of 2404 UK Healthcare Workers
Source: Antibiotics (Basel). 2022 Aug 21;11(8):1133. doi: 10.3390/antibiotics11081133 (PMC9404832; doi:10.3390/antibiotics11081133)
Supplement: Supplementary file 1 [file antibiotics-11-01133-s001.zip › Supplementary Material -UK- Launch of ECDC Survey - Comms document -.pdf]

Public Health England

Protecting and improving the nation's health

Launch: Survey of healthcare workers knowledge and attitudes about antibiotic use and resistance

On 28 January 2019, an ECDC-funded* survey to assess healthcare workers' knowledge and perceptions about antibiotic use and resistance launched across Europe. Previous studies have mostly focused on the general public and medical students, highlighting a gap in the understanding of these topics by healthcare workers and other healthcare students.

Following a process of validation and piloting across Europe, the survey is now available for completion: **https://surveys.phe.org.uk/TakeSurvey.aspx?SurveyID=9lKJ5585H**

The survey closes on **14 February 2019.**

The survey provides a good opportunity for us to have comprehensive data on healthcare workers and students' knowledge and perceptions on antibiotic use and resistance for the first time in the UK.

The aim is to have a return of 10,000+ responses with representation from healthcare workers including doctors, nurses, midwives, dentists, pharmacists, clinical scientists, hospital managers, allied health professionals, nursing associates, technicians and healthcare students across Europe.

The objectives of the survey for ECDC are:

- to gain a better understanding of their knowledge and perceptions to provide a base to support future needs in terms of policy and education changes, and
- to fill in gaps in terms of evaluation of communication campaigns targeting healthcare workers

Please cascade the link of the survey actively to relevant organisations and colleagues as well as healthcare students. If you are using social media to promote please add **#ECDCAntibioticSurvey** to your messages. For questions about this survey email espaur@phe.gov.uk.

Thank you very much in advance.

Dr Diane Ashiru-Oredope
Project Lead; Europe wide Survey of healthcare workers knowledge and attitudes about antibiotics and antibiotic resistance
espaur@phe.gov.uk

* European Centre for Disease Prevention and Control

#ECDCAntibioticSurvey

Notes:
PHE was commissioned by ECDC in September 2018 following a competitive procurement bid.

The final survey has been developed following a validation process by a diverse Project Advisory Group which includes colleagues from ECDC, WHO-Europe, country focal points as well as representatives from EU level professional organisations for example EU-JAMRAI, ESCMID/ESGAP, Council of Dentists, Pharmaceutical Group of the European Union, European Midwives Association, European Specialist Nurses Organisations, and the Standing Committee of European Doctors.

The pilot survey was completed by healthcare workers across Europe with further edits based on comments provided.
